# High-THC *Cannabis sativa* in a New York greenhouse: yield and economic factors

**DOI:** 10.1186/s42238-026-00429-5

**Published:** 2026-05-06

**Authors:** Daniela Vergara, Kayla A. Ruterbories

**Affiliations:** 1 Cornell Cooperative Extension, Harvest NY, Geneva, NY 14456 USA; 2 11929 Warner Gulf Rd, East Concord, NY 14055 USA

**Keywords:** Autoflower vs. photoperiod, Greenhouse production economics, Labor costs, Marijuana, Profitability, Yield optimization

## Abstract

**Background:**

The legalization of adult-use *Cannabis sativa* in New York State has created a need for research-based information on expected yield, production costs, revenue, and profitability for greenhouse cultivation. Limited data currently exist to inform growers and investors. This study evaluates both agronomic and economic outcomes for two flowering strategies—autoflower (light-insensitive) and photoperiod (light-sensitive) *C. sativa*—grown in a NYS greenhouse.

**Methods:**

A comparative agronomic and economic analysis was conducted to assess yield performance, input requirements, costs, revenue, and returns for autoflower and photoperiod *C. sativa* crops. Growth traits were measured and correlated with final yield. Cost components, including labor, seeds and plants, nutrients, and other variable inputs, were analyzed to determine their contribution to total production expenses. Economic returns were calculated on a per–square foot basis.

**Results:**

Both autoflower and photoperiod plants showed strong correlations between early growth traits and final yield. Autoflowers, with shorter life cycles and independence from light manipulation, produced smaller plants with lower total biomass and THC content compared to photoperiod plants. Using assumed baseline values, autoflower cultivation resulted in a negative annual return above total costs of negative $1.48 per ft^2^, whereas photoperiod cultivation generated a positive return of $7.18 per ft^2^. Labor represented the largest share of variable costs for both systems, accounting for 52% of total costs in autoflower production and 34% in photoperiod production.

**Conclusions:**

Autoflowers may be advantageous in space, capital, or labor-constrained environments requiring rapid crop turnover, while photoperiod plants appear more profitable for larger or well-resourced operations focused on maximizing yield and returns. Additional research is needed to identify practices and economic strategies that improve profitability, consistency, and efficiency for both cultivation approaches. This study underscores the need for continued economic analyses to guide decision-making in the emerging adult-use *C. sativa* industry.

**Supplementary Information:**

The online version contains supplementary material available at 10.1186/s42238-026-00429-5.

## Background

*Cannabis sativa* L. (marijuana, hemp) is one of the earliest domesticated crops (Li 1973, 1974; Russo 2007) and belongs to the angiosperm family Cannabaceae (Bell et al. 2010). Marijuana-type *C. sativa* produces cannabinoids with medicinal (Russo 2011; Swift et al. 2013; Volkow et al. 2014) and psychoactive properties (ElSohly and Slade 2005; Russo and McPartland 2003). The dominant psychoactive molecule, THCA, decarboxylates to THC when heated (ElSohly and Slade 2005; Russo and McPartland 2003); THC content strongly influences market value (Smart et al. 2017; Dobbins et al. 2022) and is tightly regulated (ElSohly and Slade 2005; Russo and McPartland 2003). Flower quality, which includes cannabinoid concentration, is shaped by genotype, morphology, and post-harvest handling (Lapierre et al. 2023; Sandhu et al. 2022; Punja et al. 2019, 2023).

### Plant biology and agronomic factors

*Cannabis sativa* is photosensitive, with flowering normally triggered by shortening daylength; however, autoflowering varieties flower independently of photoperiod and often remain smaller, making them suitable for low-light-control environments (Kurtz et al. 2023; Toth et al. 2022). Across varieties, biomass yield relates strongly to plant morphology: wet and dry biomass are linearly related, stem diameter is the best predictor of final dry floral mass, and smaller, early-flowering plants have higher dry-to-wet ratios though not necessarily greater yield per area (Carlson et al. 2021). Processing can reduce biomass by 25–77% (Warner et al. 2017), and inflorescence structure, canopy density, and plant section strongly influence cannabinoid distribution (Danziger and Bernstein 2022; Stack et al. 2023). Phenotypic diversity in vigor and growth rate is linked to floral productivity (Naim-Feil et al. 2021), and cannabinoid yields vary by genotype and developmental stage (Burgel et al. 2020). Environmental and management practices, which include transplant timing, spacing, and plant density, strongly affect morphology and biomass distribution (Naim-Feil et al. 2021; Linder et al. 2022).

### Global and regional economics

The global *C. sativa* economy continues to expand, with legal adult-use markets projected to reach $64.73 billion in 2024 and grow to $75.09 billion by 2029 (Department SS 2023a, 2021, 2023b). North America remains the dominant driver of this growth. In 2023, legal U.S. revenues reached $28.8 billion, rising from $26.1 billion the previous year, with average monthly sales of $2.4 billion and year-over-year growth of 10.3% (Whitney 2024a; Barcott et al. 2024). Consumer demand is shifting toward a broader range of products, and processed and manufactured goods now represent 45–50% of total retail revenue (Whitney 2024a).

Nationally, adult-use markets are influenced by evolving consumer preferences, regulatory changes, and broader macroeconomic forces (CFAH 2024). *Cannabis sativa* use continues to rise: 17% of Americans reported smoking marijuana in 2024, and 50% of U.S. adults report having tried it, compared to just 4% in 1969 (Gallup 2024). Product preferences vary across generations, with flower and pre-rolls making up roughly half of sales for most age groups, while Generation Z shows a stronger shift toward manufactured products (Whitney 2024a). These national consumption patterns highlight the need for diverse production and cultivation strategies, including both photoperiod and autoflower varieties, to meet a rapidly diversifying market.

In New York State, the adult-use market has accelerated quickly despite early delays caused by litigation and a complex social-equity-focused licensing rollout. Legal sales totaled $264 million in 2023 and reached $651 million by September 2024, putting the market on track to approach $1 billion annually (Christmann 2024; Whitney 2024b). New York City is the world’s largest marijuana-consuming city, with 62.3 metric tons used annually at an average price of $12.50 per gram (CFAH 2024). Consumer behavior mirrors national trends: in 2021, 12.8% of adults—about 1.6 million people—reported past-30-day use, with smoking the most common mode of consumption (BRFSS 2021). The industry created more than 22,000 new jobs in 2023 (Barcott et al. 2024), underscoring emerging workforce needs as the state’s market matures and stabilizes.

### Need for economic analyses

Growers require research-based economic information to assess the viability of adult-use *C. sativa* enterprises, including whether to cultivate photoperiod or autoflower varieties, which differ in labor, plant size, and production timelines. Although reviews of hemp fiber and grain economics are available (Mark et al. 2020; Mark and Will 2019; Kim and Mark 2023), comparable analyses for high-cannabinoid adult-use production remain limited due to recent legalization. Labor has been identified as the largest variable cost in *C. sativa* cultivation (Hanchar et al. 2022). This study expands on previous work by incorporating newly collected farm-level data from a New York State greenhouse to evaluate costs, revenues, returns, and sensitivity scenarios relevant to modern adult-use production systems.

## Methods

Plants were grown in a controlled greenhouse environment with measured light energy of 51,574 J/cm^2^, recorded by the facility’s environmental monitoring system. An 18:6 h light:dark photoperiod was maintained during the vegetative stage and 12:12 h during flowering. Average air temperature was 22 °C, relative humidity approximately 54%, and CO₂ concentration 1,400 ppm. Irrigation was applied via an automated system delivering one 15 mL pulse per event, adjusted based on weather conditions and light availability, using a proprietary multi-nutrient solution. Plants were spaced 3 ft (~ 1 m) apart and grown in 3-gallon grow bags filled with coco coir. Greenhouse structure, glazing, shading, heating, cooling, and ventilation followed standard operational practices.

### Autoflowers

**Fig. 1:**
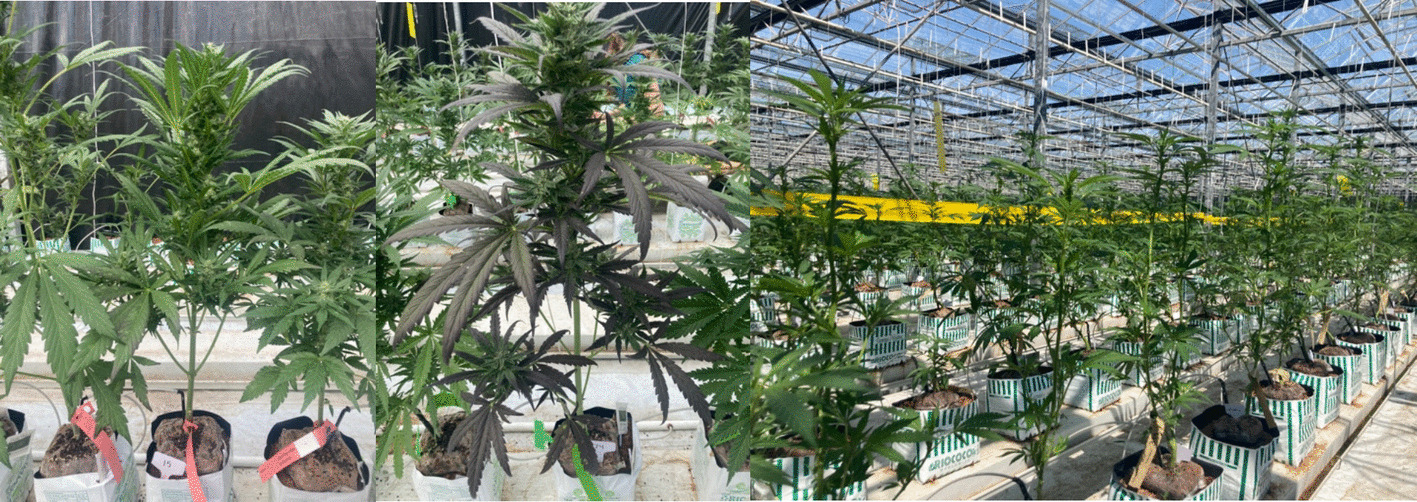
Pictures of the different varieties measured, the two autoflowers Caramel Cream Gelato (left), and Sour Apple (center), and one of the photoperiod varieties Bop Gun (right)

Autoflower, or light-insensitive, plants from the varieties “Sour Apple” and “Carmel Cream Gelato” were cultivated between February and May 2023 (Fig. 1). Seeds were started in mid-February, and plants were transplanted to their final location on March 6, allowing a 60-day growth period in the greenhouse. A total of 100 plants, 50 from each variety, were assessed at four intervals throughout the season and at harvest, totaling five measurement points. For each timepoint, three growth metrics were recorded: plant height (cm), stem diameter (mm), and node count. At the May 4 harvest, additional measurements were taken, including the size and width of the largest inflorescence (cm and mm, respectively) and the wet mass of each plant (g) including the root mass. Twelve days post-harvest, on May 16, dry mass (g) was recorded for 49 of the 100 plants.

This comprehensive measurement approach provided insights into growth patterns and final yield across both varieties. The study’s structured data collection at multiple stages allowed for analysis of developmental differences within the two autoflower varieties under consistent growing conditions.

### Photoperiods

Photoperiod-sensitive plants from six varieties—"Bop Gun," "Doc Holiday 4," "GMO," "Animal Face," "Kosher Kush," and "Donkey Butter"—were cultivated from April to June 2023 (Fig. 1). The first batch of seeds was started in late March, transplanted to the greenhouse on April 3, and grew for 85 days until harvest on June 26. A second batch of "Bop Gun," "Doc Holiday 4," and "Kosher Kush" was started two weeks later and transplanted on April 14, resulting in 74 days of field growth. In total, 90 plants, with 10 from each variety across both planting batches, were assessed at two timepoints during the growing season and at harvest. Each measurement period included the same three metrics: height (cm), stem diameter (mm), and node count.

At harvest, additional data were collected, including the size and width of the largest inflorescence, as well as the wet mass of the entire plant (g) and the wet bucked mass (g) after the removal of leaves and stems, leaving the usable biomass.

### Cannabinoid testing

Cannabinoid testing for 12 cannabinoids, covering both acidic and neutral forms and including THC, was conducted through third-party laboratories selected by the company from which the plant material and production data were obtained. The company provided the resulting cannabinoid measurements for both photoperiod and autoflower *C. sativa* plants. Cannabinoid quantification was carried out using high-performance liquid chromatography (HPLC). Analyses were performed on dried female inflorescences, the plant tissues where cannabinoid concentrations are highest (Potter 2004, 2009).

### Statistical analyses

T-tests were conducted to evaluate differences in the shared traits measured between autoflower and photoperiod plants, including wet plant mass (g), diameter of the main inflorescence (mm), size of the main inflorescence (cm), number of nodes, stem diameter (mm), height (cm), and number of days on the ground.

Additionally, for the photoperiod plants, a linear mixed-effects model analyzed the fixed effects of timepoint, age, and their interaction, with plant ID as a random effect for repeated measures. F-tests evaluated the fixed effects, and post-hoc age comparisons were performed using Tukey's method. This analytical approach provided a detailed view of growth variation across photoperiod-sensitive plants and allowed for in-depth comparison of yield potential between the two planting ages.

All statistical analyses were conducted in the R statistical platform using the packages dplyr (Wickham H: dplyr 2015), tidyr (Wickham 2025), PerformanceAnalytics (Peterson et al. 2020), doBy (Højsgaard et al. 2013), and ggplot2 (Wickham 2016).

### Economic analysis

Enterprise budgeting concepts provide the general framework for the economic analysis (Hanchar et al. 2022; Kay 1986). USDA’s Hemp Report provides price received and production information, with definitions of key items (USDA/NASS, 2024). Detailed activity analysis, with an emphasis on tracking labor and other inputs, was conducted in 2023 to generate input data for the analyses of greenhouse cultivation systems for autoflower and photoperiod *C. sativa* plants. This analysis focused on a well-equipped 30,000 sq. ft. (2,782 sq. m) greenhouse consisting of ten 3,000 sq. ft. (279 sq. m.) bays, with autoflower plants grown in one bay and photoperiod plants grown in the remaining nine bays.

Labor hours were recorded by task and by day from initial planting through harvest and final on-farm processing (bucked, dried flower) for both cultivation types. Autoflower plants were grown from seeds over approximately 62 days, resulting in about 450 plants harvested from one bay. Photoperiod plants, grown from purchased clones over approximately 91 days, produced about 3,150 plants harvested across nine bays. For comparison, annual expected costs, revenues, and returns were calculated assuming full use of the 30,000 sq. ft. (2,782 sq.m) growing space, with five autoflower and four photoperiod grows annually. Results reflect a year of activity, reported as $ per 30,000 sq. ft. (2,782 sq. m.) and $ per sq. ft.

### Use of Artificial Intelligence Tools

Portions of this manuscript were drafted and revised with assistance from OpenAI’s ChatGPT (GPT-5, September 2025). The authors reviewed and edited all AI-assisted text and take full responsibility for the final content. ChatGPT was used to improve clarity, conciseness, and flow of the writing and was not used to generate or analyze data, perform statistical analyses, or draw scientific conclusions. Both ChatGPT and Claude Opus 4.5 were used to confirm the economic analysis.

## Results

### Autoflowers

**Fig. 2:**
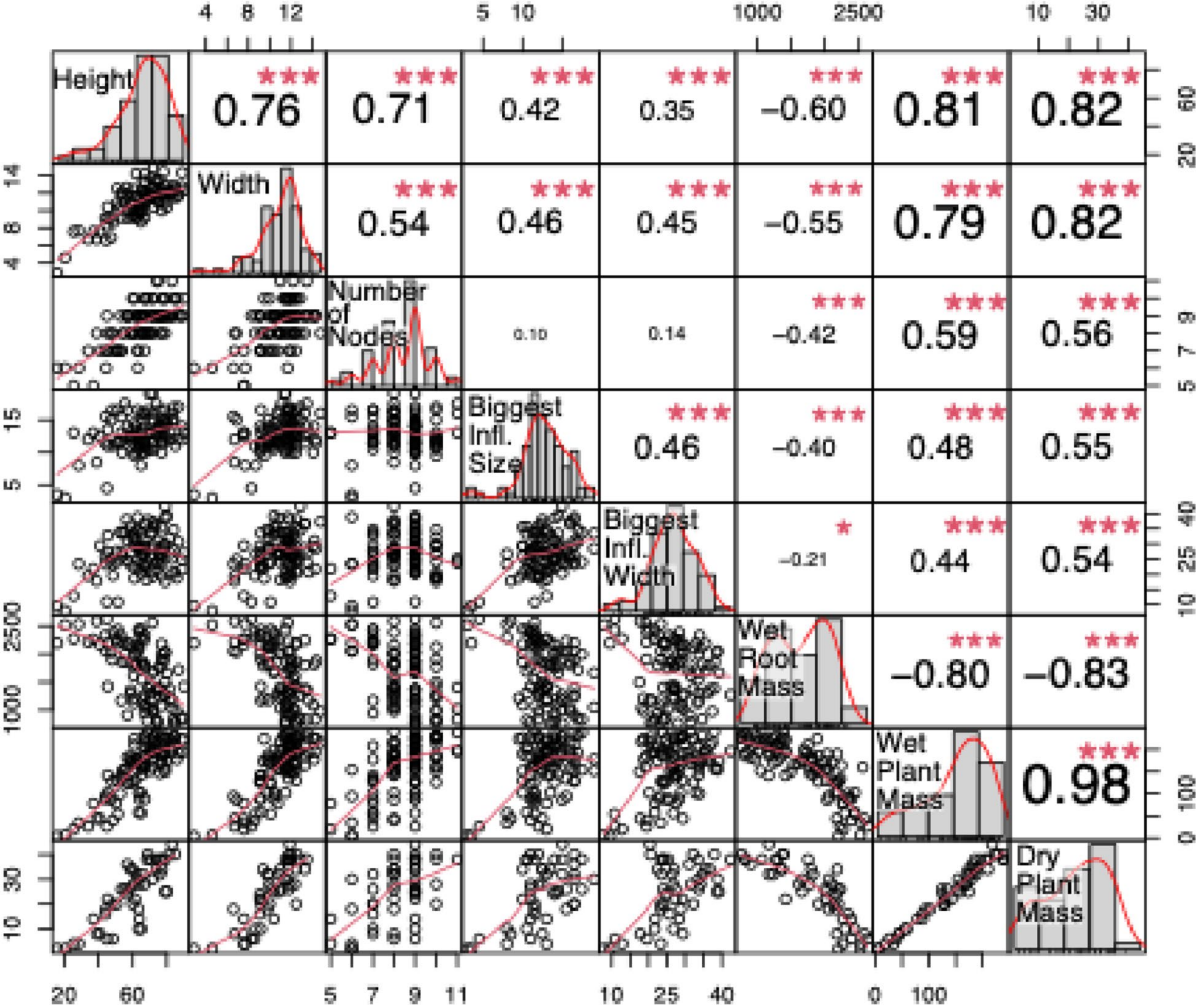
Pairwise relationships among eight harvest-stage traits measured in autoflower plants. Each diagonal panel shows the distribution of an individual trait using a histogram overlaid with a density curve. The panels below the diagonal display bivariate scatterplots with a fitted smoothing line to illustrate the form of each relationship. The panels above the diagonal report the Pearson correlation coefficients together with their significance levels. Significance is represented by symbols: *** (*p* ≤ 0.001), ** (*p* ≤ 0.01), * (*p* ≤ 0.05),.(*p* ≤ 0.1), and a blank space for nonsignificant values (*p* > 0.1). Together, the matrix summarizes the strength, direction, and statistical support of associations among all measured traits

The traits measured, height (cm), stem diameter (mm), node count, size (cm) and width (mm) of the largest inflorescence, the wet mass (g) and the bucked mass (g), tend to be correlated. Therefore, those plants that are tall, have numerous nodes, and a thick stem diameter (Fig. 2). Additionally, traits are correlated among times, therefore those plants that are tall when young are tall when old (Figure S1). The mean and standard deviation for the traits measured during harvest are given in Table 1.

**Table 1:** Measurements during harvest for six traits shared among both autoflower and photoperiod plants showing mean ± standard deviation (columns 3–8); the dry plant mass collected only for autoflower plants (column 9); the wet bucked mass collected only for photoperiod plants (column 10); the Total THC provided through third-party testing (column 11), and estimates in italics of the effective mass (column 12) and the estimated THC mass and range (column 13)

		**Mean ± Standard Deviation**										**Estimates**	
**Variety**	**Flowering Strategy**	**Height (cm)**	**Width (mm)**	**Number of Nodes**	**Biggest Inflorescence Size (cm)**	**Biggest Inflorescence Width (mm)**	**Wet Plant Mass (g)**	**Dry Plant Mass (g)**	**Wet Bucked Mass (g)**	**Total THC (mg/g)**	**Total THC (mg/g)**	***Effective Mass (g) mean* ± *s.d***	***THC Per Plant (g) -range***
Caramel Cream Gelato	A	62.82 ± 15.46	11.04 ± 2.09	8.02 ± 1.12	12.94 ± 2.57	28.17 ± 6.59	146.96 ± 60.60	19.83 ± 11.36	NA	98.45		*19.76* ± *8.15*	*1.95 (1.14—2.75)*
Sour Apple	A	66.26 ± 15.06	10.80 ± 2.09	8.92 ± 1.19	12.81 ± 3.09	25.78 ± 6.87	167.76 ± 61.89	28.62 ± 11.19	NA	99.83		*22.56* ± *8.32*	*2.25 (1.42—3.08)*
Animal Face	P	138.85 ± 10.46	20.68 ± 3.08	16.20 ± 1.39	10.00 ± 1.18	11.93 ± 3.39	844.30 ± 256.00	NA	554.87 ± 158.63	150.3		*113.54* ± *34.47*	*19.8 (13.8—25.79)*
Bop Gun	P	112.10 ± 21.06	20.17 ± 3.11	13.00 ± 2.20	12.98 ± 3.23	15.24 ± 7.04	561.60 ± 203.33	NA	397.25 ± 141.04	NA		*75.52* ± *27.34*	*NA*
Doc Holiday 4	P	87.02 ± 13.09	15.35 ± 8.40	12.59 ± 1.27	11.95 ± 3.16	16.20 ± 4.08	416.50 ± 122.40	NA	345.00 ± 99.55	NA		*56.01* ± *16.46*	*NA*
Donkey Butter	P	129.00 ± 10.93	19.26 ± 1.72	13.78 ± 1.09	11.17 ± 1.58	19.14 ± 5.29	630.56 ± 236.89	NA	414.47 ± 153.11	168.7		*84.8* ± *31.86*	*14.31 (8.93—19.68)*
GMO	P	153.70 ± 12.75	21.52 ± 2.74	17.60 ± 2.37	10.65 ± 2.3	11.09 ± 4.64	967.76 ± 245.22	NA	634.92 ± 92.44	179.8		*130.14* ± *32.98*	*23.4 (17.47—29.33)*
Kosher Kush	P	158.58 ± 18.84	20.10 ± 2.58	15.85 ± 1.59	12.25 ± 2.32	18.12 ± 5.15	765.65 ± 253.04	NA	524.37 ± 185.82	149.7		*102.96* ± *34.03*	*15.41 (10.32—20.51)*

Using linear mixed-effects models for repeated measures analysis, significant changes over time were observed in autoflower plants for height (F = 1075.3; *P* < 0.0001), stem diameter (F = 1081.7; *P* < 0.0001), and number of nodes (F = 483.58; *P* < 0.0001; Figure S1). However, the length and width of the main inflorescence did not differ by variety at harvest for autoflowers.

### Photoperiods

**Fig. 3:**
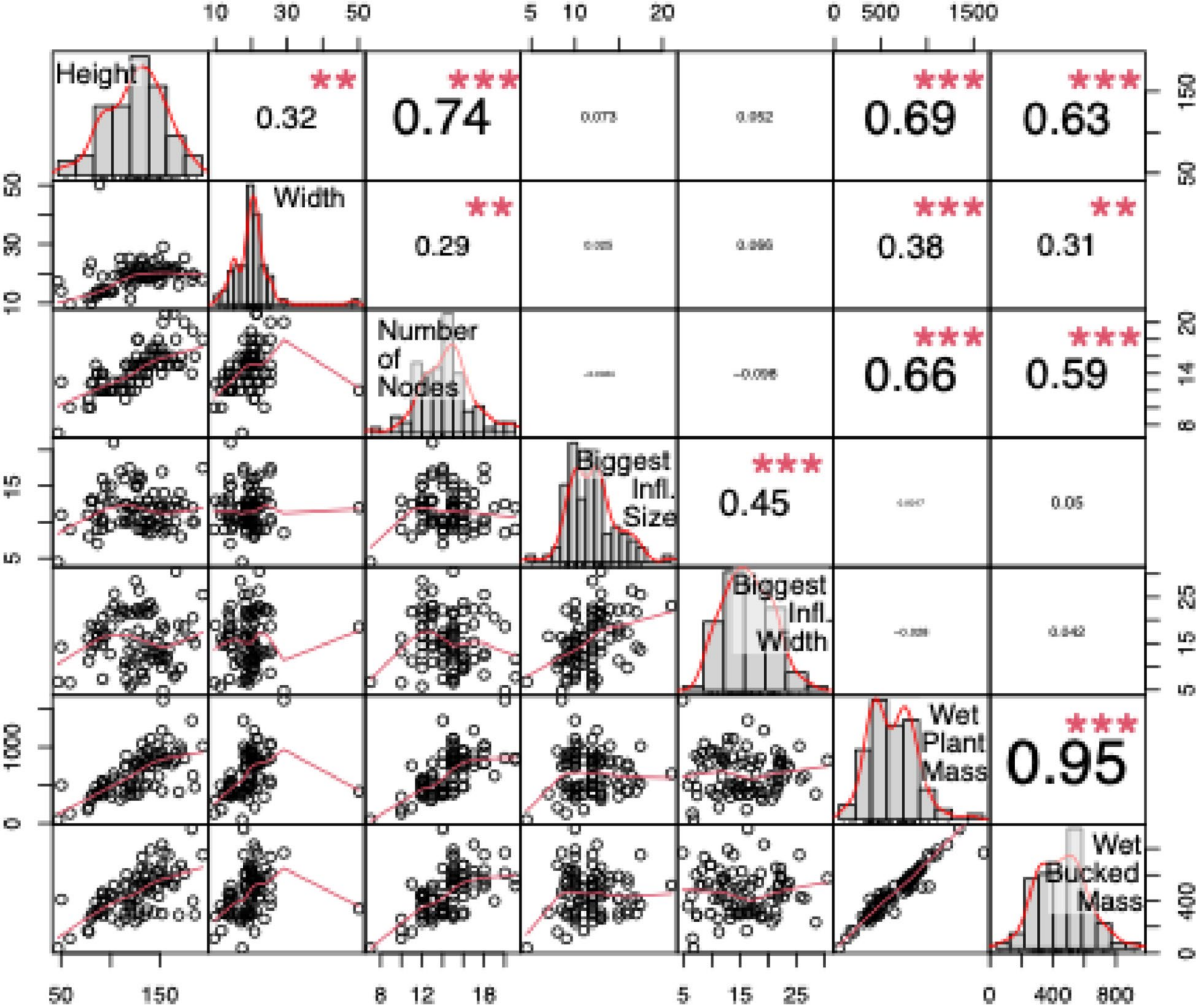
Pairwise relationships among the seven harvest-stage traits measured in photoperiod plants. Each diagonal panel displays the distribution of an individual trait using a histogram with an overlaid density curve. The panels below the diagonal show bivariate scatterplots with fitted smoothing lines to illustrate the form and direction of each relationship. The panels above the diagonal report the Pearson correlation coefficients together with their statistical significance. Significance levels are denoted as follows: *** (*p* ≤ 0.001), ** (*p* ≤ 0.01), * (*p* ≤ 0.05),. (*p* ≤ 0.1), and a blank space for nonsignificant values (*p* > 0.1). Collectively, the matrix summarizes the strength, pattern, and statistical support for trait associations in photoperiod plants

Like the autoflowers, most traits (height (cm), stem diameter (mm), node count, size (cm) and width (mm) of the largest inflorescence, wet plant mass (g), and wet bucked mass (g)) are almost always correlated (Fig. 3). Therefore, those plants that are tall, have numerous nodes, and a thick stem diameter. Additionally, traits are correlated among times. Therefore, those plants that are tall when young are tall when old (Figure S2). The mean and standard deviation for the traits measured during harvest are given in Table 1.

Using different linear mixed-effects models that allow for repeated measures analysis, in photoperiods height (F = 477.36; *P* < 0.0001), stem diameter (F = 170.44; *P* < 0.0001), and number of nodes (F = 116.88; *P* < 0.0001) all exhibited significant changes over time (Figure S2).

The only variety whose size of main inflorescence differed at harvest was “Animal Face” which was significantly smaller than “Bop Gun”. The inflorescence diameter at harvest from the variety “Animal Face” was marginally smaller from Bop Gun”, all other varieties didn’t differ in their width.

The age difference between those three varieties -the ones that were planted two weeks before and therefore had 11 days more on the ground- made no difference in the last point during harvest except for three traits (Figure S3). In other words, there were statistically significant differences between the measured traits that got smaller as the plants aged, and therefore the differences at timepoint one are larger than timepoint three.

At harvest, the mean wet plant mass per photoperiod plant was approximately 659.22 ± 277.24 g, resulting in a total of 58,670.6 g (58.67 kg) for the 89 plants sampled. The mean wet bucked mass, which excludes stems and other non-essential parts, was approximately 459.5 ± 170.03 g per plant. For the 89 plants measured, the total wet bucked mass was 40,436.1 g (40.44 kg).

### Mass correlations

**Fig. 4:**
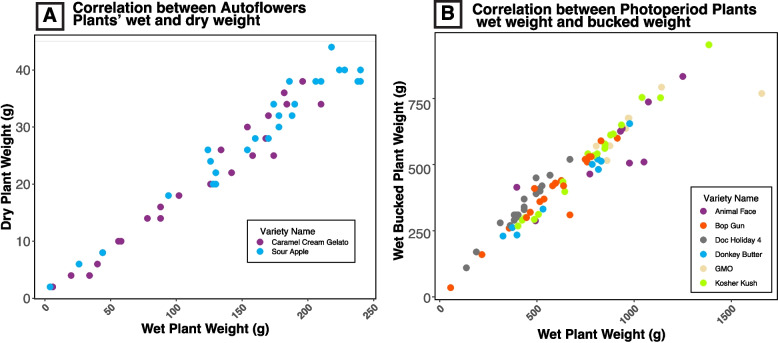
Correlation between plant mass for different *C. sativa* varieties. **A** A positive correlation is observed between wet mass and dry mass for two autoflower varieties (*P* < 0.0001, r = 0.99; Y = 0.31 + 0.17X). **B** A positive correlation is noted between wet mass and wet bucked mass for six photoperiod varieties (*P* < 0.0001, r = 0.95;Y = 78 + 0.58X)

A positive correlation was identified between the wet mass and dry mass of autoflower plants for both Sour Apple and Caramel Cream Gelato varieties (*P* < 0.0001, r = 0.98, Fig. 4A). At harvest, the average wet mass per plant was 157.36 ± 61.83 g, and the average dry mass was 24.49 ± 12 g, with a total dry mass of 1,200 g for all plants. On average, 18.6% of the mass remained after drying, indicating an ~ 82% mass loss during the drying process. The mass per autoflower variety are given in Table 1.

There is a positive correlation between the photoperiods plant’s wet mass and plant’s wet bucked mass for both all photoperiod varieties (*P* < 0.0001, r = 0.95, Fig. 4B). On average, 72% of the mass remains (~ 28% is lost) after the plant is bucked. The mass per photoperiod variety are given in Table 1.

### Autoflowers vs Photoperiods

There is a significant difference in all measured traits between autoflowers and photoperiods, as well as in the number of days on the ground (Table 2).

**Table 2:** Comparison of the various traits measured in both autoflower and photoperiod plants, including the p-value and mean for each flowering strategy

	Wet plant mass (g)	Diameter of main inflorescence (mm)	Size of main inflorescence (cm)	Number of Nodes	Stem diameter (mm)	Height (cm)	Number of Days on Ground
*P* value	< 0.0001	< 0.0001	< 0.0001	< 0.0001	< 0.0001	< 0.0001	< 0.0001
Mean autoflowers	157.36	26.98	12.88	8.47	10.92	64.54	59.76
Mean Photoperiods	659.22	15.66	11.80	14.50	19.17	126.30	80.11

### Estimates for autoflowers and photoperiods

From the autoflower analysis, approximately 82% of the plant's mass is lost due to water loss, leaving 18% as the remaining mass. In the photoperiod analysis, about 28% of the mass is lost after accounting for the removal of stems and twigs, with 72% of the mass remaining. Using these percentages, we calculated the effective mass—the usable plant material (Table 1, Column 12). On average, the effective mass is 21.16 ± 8.32 g for autoflowers and 88.65 ± 37.28 g for photoperiods. Additionally, we estimated the harvest index for both plant types (Figure S4). Considering the combined losses from water, stems, and twigs, approximately 13.5% of the plant's wet mass at harvest remains as usable material (Fig. 4).

For the 100 autoflowers grown, the mean mass was 157.36 g and their total mass was 1,573.6 g (1.57 kg). Their estimated effective mass would be 2,116.15 g (2.12 kg). If 450 autoflower plants were grown, their total wet mass at harvest would be an estimated 70,812 g (7.08 kg) and a projected effective mass of around 9,522.17 g (9.52 kg).

The average THC concentration for the autoflower varieties was determined to be 99.13 mg/g, based on Sour Apple (98.45 mg/g) and Caramel Cream Gelato (99.83 mg/g Table 1 column 11). Using this average THC potency, an autoflower plant is estimated to produce approximately 2.1 g of THC (Table 1, Column 13). For the projected effective mass of 9,521.66 g (9.52 kg) from 450 autoflower plants, the total THC yield is estimated to be 943.98 g (0.94 kg).

For the 89 photoperiod plants analyzed, the mean wet mass is 659.22 g, with a total wet mass of 58,670.6 g (58.67 kg). This corresponds to an estimated effective mass of 7,889.91 g (7.89 kg) and an estimated THC production of 1,279.15 g (1.25 kg). Scaling this to a scenario with 3,150 plants, the estimated total wet mass would be 2,076,544 g (2,076.54 kg), with an effective mass of 279,249.5 g (279.25 kg). The total THC produced by these 3,150 plants is projected to be 45,273.32 g (45.27 kg). THC estimates for individual varieties are provided in Table 1, column 13.

### Economics of autoflowers

Based upon available data, and for the median expected price received, yield combination – 260 ($ per lb. harvested floral, dried; $573 per kg), and 0.04 (lbs. harvested floral, dried per plant; 0.018 kg) – estimated value of production, variable input cost, total cost, and return above total costs total $7.80 ($84.03 per sq m), $5.58 ($60.07 per sq m), $9.29 ($99.98 per sq m), and negative $1.48 per sq. ft., (negative $15.96 per sq m.) respectively (Table 3). Total values, and calculations reflect results of rounding. Cost values represent the value of the input(s) used in production. Since total costs exceed value of production (revenue), subtracting $9.29 from $7.80 yields a negative return above total costs, or negative $1.48 per sq. ft (negative $15 per sq m). Expressed in annual $ for the 30,000 sq. ft. (2,782 sq. m.) facility, the return is negative $44,510. Total annual costs of $278,510 for the facility exceed the value of production, revenue of $234,000. The result is a return, profit value that is less than zero. Sensitivity analysis suggests that 3 of 9 output price, yield combinations produced positive returns above total costs annually (Table 4).

**Table 3:** Annual value of production (revenue), costs and returns for high cannabinoid *C. sativa* cultivation, greenhouse (under protection) setting, by planting scenario (flowering strategy). These analyses are based on the following assumptions: price received is taken from the median point of the expected range at $260 per lb. ($573 per kg), yields assumed are 0.04 lbs (0.018 kg) and to be 0.18 lbs (0.082 kg). per plant for autos and photos, respectively, and the cost of hired labor is set at $20 per hour, as outlined in the methods section. Price per sq m given in parenthesis

		**Planting Scenario**		
	Autoflower		Photoperiod	
	$/30,000 sq. ft (2,782 sq. m.)	$/sq. ft ($/sq. m)	$/30,000 sq. ft. (2,782 sq. m.)	$/sq. ft ($/sq. m)
Value of Production (Revenue)				
Value of harvested floral, dried	234,000	7.80 (83.96)	655,200	21.84 (235.09)
Costs of Production				
Variable inputs				
Fertilizer & lime	24,500	0.82 (8.83)	19,600	0.65 (7.00)
Seeds & plants	46,650	1.56 (16.79)	185,000	6.17 (66.41)
Sprays, bios, other variable crop inputs	8,370	0.28 (3.01)	7,360	0.25 (2.69)
Labor	86,490	2.88 (31.00)	110,860	3.70 (39.83)
Interest on operating capital	1,380	0.05 (0.54)	5,380	0.18 (1.94)
Total variable inputs				
	167,390	5.58 (60.06)	328,200	10.95 (117.87)
Fixed inputs				
Land charge	140	0.01 (0.11)	140	0.01 (0.11)
Buildings, improvements, and mechanicals	89,140	2.97 (31.97)	89,140	2.97 (31.97)
Value of operator & family management	14,850	0.50 (5.38)	14,850	0.50 (5.38)
Other fixed inputs	6,990	0.23 (2.48)	7,470	0.25 (2.69)
Total fixed inputs				
	111,120	3.71 (39.93)	111,600	3.73 (40.15)
Total costs				
	278,510	9.29 (100.00)	439,800	14.68 (158.02)
Returns				
Revenue minus costs of variable inputs	66,610	2.22 (23.90)	327,000	10.90 (117.33)
Revenue minus costs of variable & fixed inputs	−44,510	−1.48 (−15.93)	215,400	7.18 (77.29)

**Table 4:** Revenue less total costs by price and yield for high-cannabinoid *C. sativa* cultivation in a greenhouse setting (30,000 sq. ft.; 2,782 sq. m.). The first three columns represent autoflower planting scenarios with five two-month cycles annually, and the last three columns represent photoperiod planting scenarios with four three-month cycles annually. Kg values given in parenthesis

	**lbs. (kg) floral, dried per plant**					
	**Autoflowers**			**Photoperiods**		
$ per lb. floral, dried ($ per kg)	0.02 (0.009)	0.04 (0.018)	0.06 (0.027)	0.11 (0.50)	0.18 (0.82)	0.25 (0.113)
120 (265)	−224,510	−170,510	−116,510	−255,000	−137,400	−19,800
260 (573)	−161,510	−44,510	72,490	−39,400	215,400	470,200
400 (882)	−98,510	81,490	261,490	176,200	568,200	960,200

### Economics of photoperiods

Based upon available data, and for the median expected price received, yield combination – 260 ($ per lb. harvested floral, dried; $573 per kg), and 0.18 (lbs. harvested floral, dried per plant; 0.082 kg per plant) – estimated value of production, variable input cost, total cost, and return above total costs total $21.84 ($235.08 per sq m), $10.95 ($117.87 per sq m), $14.68 ($158.02 per sq m), and $7.18 per sq. ft. ($77.29 per sq m), respectively (Table 3). Since the value of production exceeds the total cost of production, return is greater than zero. Sensitivity analysis suggests that 5 of 9 output price, yield combinations produce positive returns above total costs annually (Table 4).

## Discussion

Our results show that autoflower plants are smaller than photoperiod plants and produce less mass, including lower THC content per gram of flower in the two autoflower varieties studied (Tables 1 and 2). The mass loss from wet to dry in autoflower plants, approximately 82%, was higher than the 77% previously reported (Warner et al. 2017). Additionally, the photoperiod analysis suggests that the mass from stems and twigs accounts for about 18% of the wet mass. The positive correlations between various traits in both autoflower (Fig. 1) and photoperiod (Fig. 2) plants indicate that early plant characteristics can predict final size and yield. Because autoflowers have shorter growth cycles and flower independently of light, they may present a cost-effective option for indoor or greenhouse cultivation where available capital and labor are limited. These plants require less time in the ground and demand minimal pruning or trellising (Vergara et al. 2023), which can reduce labor and costs. However, in controlled environments where light cycles can be easily adjusted, photoperiod plants may be manipulated to flower at smaller sizes and for shorter periods, offering flexibility in production. Another drawback of autoflowering plants is their lack of consistency, often attributed to poor breeding practices. However, this inconsistency has not been thoroughly quantified or directly compared with photoperiod plants, leaving it largely speculative.

Our results indicate that, beyond the differences observed between autoflower and photoperiod plants, significant variation exists within varieties of each flowering strategy in terms of THC content, yield, and biomass production.

Previous estimates on cannabinoid production, specifically CBD (Stack et al. 2021), suggest higher yields compared to the estimated THC production observed in the varieties measured here. However, the THC yields reported in this study fall within these previous estimates (Stack et al. 2021). It is important to note that the previously reported values were derived from plants grown outdoors, many of which spent over 85 days in the ground (Stack et al. 2021), allowing them to grow larger. The measured cannabinoid in those studies was CBD, not THC, which introduces another important difference and limitation when comparing these results.

### Cultivation practices that can affect quality and yield

In photoperiod plants it has been shown that topping did not significantly improve flower yield or cannabinoid concentration. While flower yield per plant decreased with higher plant density, total yield per hectare increased. CBD production per hectare rose with greater density, but cannabinoid concentration remained unaffected. However, increased density does not guarantee higher economic returns due to the high input costs for hemp plant material and labor (Silva Benevenute et al. 2022). Topping plants 3–4 weeks after transplanting increased labor costs without improving yield or cannabinoid content. While topping increased inflorescences and CBD content in two varieties (Folina et al. 2020), it also significantly influenced plant height, with un-topped plants being taller. Architectural modulation methods, including selective pruning and defoliation, improved cannabinoid profile consistency by reducing concentration variability across the plant's height. Yet, methods like primary branch removal reduced total yield, highlighting the challenge of balancing plant structure and cannabinoid optimization (Lapierre et al. 2023; Danziger and Bernstein 2021). As previously mentioned, these metrics are currently lacking for autoflowering plants.

### Economics of autoflower cultivation

Estimated value of production given initial price received and yield assumptions total $7.80 per sq. ft. (Table 3). Value of production estimates are a function of the number of grows per year, plants per grow, yield per plant, price received. Price received and yield variability substantially impact profit (Table 4). Results suggest that evaluating alternative practices for production and economic efficiencies are important to identifying the optimal set of production practices – planting settings, autoflowers and/or photoperiods, number and lengths of growing cycles which will differ between these two flowering strategies, number of plants per grow, among other considerations.

Variable costs ($ per sq. ft.) total 5.58 and account for 60 percent of total costs. Labor, seeds & plants, and nutrients are the three largest $ per sq. ft. items. Labor costs are the single largest item, accounting for 52 percent of total variable input costs ($ per sq. ft.). Seeds & plants expense, the second largest item, and nutrients the third largest, account for 28 and 15 percent of total variable input costs ($ per sq. ft.), respectively. These three greatest variable cost items account for 95 percent of all total variable input costs.

Total cost fixed inputs ($ per sq. ft.) total 3.71, and account for 40 percent of total costs. Fixed costs for buildings, improvements, and mechanicals for the greenhouse account for the vast majority of total fixed costs. Estimated total cost for variable and fixed inputs equals $9.29 per sq. ft., while revenue minus costs of variable inputs, and revenue minus total costs equal $2.22 and negative $1.49 per sq. ft., respectively.

### Economics of photoperiod cultivation

Estimated value of production given initial price received and yield assumptions total $21.84 per sq. ft. (Table 3). Value of production estimates are a function of number of grows per year, plants per grow, yield per plant, price received. Price and yield variability substantially impact profit (Table 4). Results suggest that evaluating alternative practices for production and economic efficiencies are important for identifying the optimal set of production practices – planting settings, auto and, or photo; number of grows; length of grows; number of plants per grow; and others.

Variable costs ($ per sq. ft.) total 10.95 and account for 75 percent of total costs. Seeds & plants, labor, and nutrients are the three largest $ per sq. ft. items. Seeds & plants expense is the single largest item, accounting for 56 percent of total variable input costs ($ per sq. ft.). Recall that for the photoperiod planting scenario, analysis reflects that purchased clones began the cultivation activities, and price paid for clones was about $13.50 per clone. Given this factor’s effect on results, future work would benefit from more accurate information regarding price paid, and or analysis of alternative practices, for example, analysis that assumes meeting the needs for clones in house. This analysis should quantify the tradeoffs between seeds & plants expense, labor, and other costs. Labor is the second largest item, and nutrients the third largest, accounting for 34 and 6 percent of total variable input costs ($ per sq. ft.), respectively. These three greatest variable cost items account for 96 percent of all total variable input costs.

Total cost fixed inputs ($ per sq. ft.) total 3.73, and account for 25 percent of total costs. Fixed costs for buildings, improvements, and mechanicals for the greenhouse account for most total fixed costs. Estimated total cost for variable and fixed inputs equals $14.68 per sq. ft., while revenue minus costs of variable inputs, and revenue minus total costs equal 10.90 and 6.58 $ per sq. ft., respectively.

### Autoflower and photoperiod economic comparison

Profit estimates for photoperiod cultivation are more favorable compared to autoflower cultivation given expected price and yield assumptions. Returns above total costs reflecting a years of activity show that the photoperiod planting scenario yielded returns above total costs of $7.18 per sq. ft., while the autoflower planting scenario yielded negative $1.48 per sq. ft. (Table 3). Sensitivity analysis results for the autoflower planting show that annual returns above total costs for a 30,000 sq. ft. facility ranged from negative $224,510 for the least favorable price received, yield combination to positive $261,490 for the most favorable price, yield combination (Table 4). Comparison values for the photoperiod planting ranged from negative $255,000 to positive $960,200, respectively. From a different perspective, sensitivity results show that for the autoflower planting, returns were greater than zero for three of the nine price, yield combinations, while the photoperiod planting produced returns greater than zero for 5 of 9 combinations. Photoperiod cultivation benefits from greater expected yields per plant, while autoflower plantings benefit from more annual grows and higher plant counts per grow. However, the net result is that photoperiod plantings annual revenue exceeds autoflower expected revenues. These higher revenues, even when combined with greater annual variable costs, drive the photoperiod scenario’s superior economic performance relative to autoflower cultivation.

Two key expense items stand out in the cost comparison: seeds and plants, and labor. For seeds, the autoflower scenario involves purchasing seeds at approximately $1.50 each for five annual grows. In contrast, the photoperiod scenario relies on clones costing nearly $13.50 each, with only four grows annually. Labor costs differ significantly. Autoflowers, requiring five grows per year with each grow lasting just over 62 days, demand less labor due to minimal need for trellising and trimming. Photoperiod plants, grown four times annually over 92-day cycles, demand more intensive trellising and trimming, increasing labor expenses.

Risk and uncertainty are prominent concerns in cultivating high-cannabinoid *C. sativa* in newly legalized markets like New York State. Fees such as licensing, applications, sampling, and testing for heavy metals, pesticides, and THC content can accumulate significantly over a growing season. Additionally, uncertainties around how these fees and taxes will be assessed add complexity and financial risk, as reflected in the variability (Tables 3 and 4).

Labor costs are a substantial component of variable input expenses, accounting for 52% in the autoflower scenario and 34% in the photoperiod scenario. Managing labor-related risks is critical due to uncertainties in labor availability, pricing, and skill levels. This analysis was strengthened by detailed tracking of labor hours by task and day, offering valuable insights for predicting labor needs and costs more accurately.

Financial risk management is essential, given the variability in economic performance influenced by price received and yield (Tables 3 and 4). Sound financial planning, including budgeting and scenario analysis, can help mitigate risks. For example, adopting best management practices can reduce the risk of low yields. Sensitivity analysis allows farm owners to evaluate the viability of their operations and identify focus areas for risk reduction, ensuring sustainable business performance amidst economic uncertainties.

### Caveats

Several limitations should be considered when interpreting these results. First, the cannabinoid analysis was conducted by a third-party laboratory, and there is evidence suggesting potential data tampering (Turkington et al. 2024; Schwabe et al. 2023). While this raises concerns about the data's reliability, we must rely on the provided analysis for this study. It is important to note that cannabinoid content can vary across different flowers of the plant (Danziger and Bernstein 2022), and in different plants from the same variety (Smith et al. 2022), yet commercial facilities typically test cannabinoids and other compounds in bulk, largely due to the high costs of testing. Additionally, the chosen varieties for this research may not represent those commonly used in other facilities, as cannabinoid content, yield, and other metrics are highly dependent on the specific variety grown. Different varieties inherently produce varying levels of cannabinoids (Smith et al. 2022) and biomass (Stack et al. 2023), which influences overall performance.

Additionally, cultivation practices play a significant role in determining yield and quality. These practices vary widely between facilities and cultivators, further complicating the generalization of these findings. Our results are based on data collected from a single facility at a specific point in time. While they provide a useful reference for guiding future cultivators and facilities in estimating production potential and costs, they may not be directly applicable to other settings. For example, we assume that photoperiod plants in this analysis are grown from purchased clones. However, plants may be grown from seeds in other facilities, or at this same facility at a different time. Differences would alter the analyses of revenue, costs, returns, and the estimates. For example, in a scenario where plants are grown from seeds, input costs for seed and plants, labor, other inputs would be expected show differences.

Additionally, autoflowering and photoperiod plants were treated differently in this study due to the distinct ways they were processed. Autoflowers were hang dried and their bucked mass wasn’t taken, while photoperiod plants were bucked while wet, and the dried mass was not measured. This discrepancy introduces assumptions that the same amount of water was lost during drying for both flowering strategies, and that the mass of stem twigs lost in both strategies is similar. As a result, the estimates provided in this study reflect the effective mass and THC content under these assumptions.

### Needs and opportunities

Policies and programs at both federal, and New York State levels create opportunities for adult-use *C. sativa* production enterprises. These enterprises offer farm business owners new crop selection options. To make informed management decisions regarding new opportunities in the *C. sativa* industry, farm business owners benefit when production, and economic research-based information are developed, available, and accessible. Industry analysts, and experts point out that farm-level agronomic, and cost and return analyses for different cultivation scenarios are still limited. Limitations make it difficult for farmers to make optimal decisions amidst the inherent risks, and uncertainties in the market. The economic analysis presented in this study seeks to provide farm-level agronomic, and economic insights into the operation of *C. sativa* enterprises.

### Approach, methods, data

Despite its limitations, our study provides estimates based on recorded, farm level, agronomic and economic data collected from a functioning farm using actual farming practices, yielding tangible products that are sold in the market. A valuable feature of this work is the comprehensive collection, recording, and analysis of labor usage, and other crop management inputs. Efforts provide actionable data for growers. This analysis benefits from a comprehensive collection, and reporting of labor hours by task, providing more informed expectations regarding labor needs, costs, and strategies for managing human resources risks.

## Results, outcomes

The approach here offers grounded insights based upon peer reviewed methods, data, results, etc. In contrast, consider other works where: 1) methods, data, and results are not accessible and, or difficult to access; and, or 2) methods, data, results etc. are omitted and, or unclear. The former approach supports improved decision making, allowing for the effective management of risks, for example, human resource risks, by way of implementing practices that mitigate risk and uncertainty.

### Future work

While this study serves as a valuable contribution to the field, there is a continued need for more production, and economic research to support farm business owners. Future studies could focus on the effects of alternative planting scenarios, the number of grows per year, plant densities, and the interactions among these factors on the overall value of production, costs, and returns. Additionally, research-based economic insights are needed to refine the understanding of price received, and yield metrics. Improved understanding ultimately empowers farm business owners to make more informed decisions about the profitability, and sustainability of their operations.

Future needs for research-based information may be addressed by way of cooperation among value chain participants, stakeholders. Perhaps a periodic survey, and reporting of key agronomic, and economic metrics via an industry supported effort could be studied for its potential to benefit the industry. *Cannabis sativa* industry value chain participants might be willing to provide data — perhaps anonymously, or in a way that does not compromise their intellectual property or operations — to a representative group of industry stakeholders charged with developing and implementing reporting activities for common use. Such collaborations could help the industry gain a clearer understanding of agronomic factors, production practices, costs, returns, and economic dynamics.

Value chain participants seek to achieve economic, environmental, and community objectives given available resources. Their efforts towards continuous improvement – What worked? What did not work, and why? – benefit from efforts to improve availability, and accessibility of research-based information. The issues related to data quantity, quality and availability; data tampering; the speculative nature of the industry; lingering illicit operations; unreliable players; and the potential for undisclosed methods, data, and assumptions of analyses concern industry value chain participants, and stakeholders. Companies that know their enterprises’ costs and production metrics hold valuable insights that could greatly benefit the *C sativa* industry as a whole, particularly in emerging markets like New York State. For example, research-based insight regarding value of production, costs, and returns associated with systems, and practices that did not achieve objectives, and goals might accelerate progress toward sustainable viability, and growth of a New York State *C sativa* industry. Periodic sharing of knowledge from, and among value chain participants, stakeholders could be transformative.

## Conclusions

This study provides one of the first farm-level agronomic and economic evaluations of high-THC *C. sativa* cultivation in a New York State greenhouse. Autoflowering plants offered shorter cycles and lower labor needs but produced smaller yields and lower profitability compared to photoperiod plants under the conditions analyzed. Photoperiod cultivation generated higher annual returns per square foot despite greater labor and cloning costs, suggesting it is better suited for operations prioritizing yield and profit optimization. However, autoflowers may still be advantageous in settings with limited capital, labor, or space, or where rapid turnover is needed. Findings highlight the importance of early growth traits for predicting final yield, the substantial role of labor and plant material in production costs, and the need for improved breeding and production data, particularly for autoflowers. Continued research on cultivation strategies, economic risks, and pricing dynamics will be essential to support informed decision-making and long-term sustainability for *C. sativa* producers.

## Supplementary Information

Supplementary Material 1. Figure S1. Changes in growth parameters over time for autoflower Cannabis sativa plants, analyzed using linear mixed-effects models with repeated measures: (A) height showed a significant change through time (F = 1075.3; P < 0.0001) and all timepoints differ from each other (P<0.0001) except timepoints 4 and 5, (B) stem diameter significantly changed through time (F = 1081.7; P < 0.0001) and all timepoints differ from each other (P<0.0001) except timepoints 4 and 5, and (C) the number of nodes exhibited significant variation over time (F = 483.58; P < 0.0001), with all timepoints differing from each other with a significance of P<0.0001 except timepoints 3 and 4 with a significance of P<0.03. Figure S2. Temporal changes in growth parameters of photoperiod C. sativa plants, analyzed using linear mixed-effects models with repeated measures: (A) height significantly increased over time (F = 477.36; P < 0.0001) with all timepoints differing from each other (P<0.0001), (B) stem diameter significantly increased over time (F = 170.44; P < 0.0001) with all timepoints differing from each other (P<0.0001), and (C) the number of nodes significantly increased over time (F = 116.88; P < 0.0001) with all timepoin. Lines in R code 2113. Figure S3. Growth and biomass traits of photoperiod C. sativa plants during harvest. The varieties planted two weeks earlier (with 11 additional days in the ground; Age 1) showed statistically significant differences during harvest in (A) height (F=8.951, P<0.001), (B) Stem diameter (F= 11.09, P<0.0001), and (C) Number of Nodes (F=6.883, P< 0.005). Figure S4. Estimated Harvest Index based on the lost mass on water from the Autoflower measurements and on the lost mass from stems and twigs after bucking from the Photoperiod measurements.

## Data Availability

Upon acceptance, the raw data supporting this study will be made publicly available through recognized online repositories, the Cornell University data repository, and the authors’ professional websites.

## Abbreviations

CBDA: Cannabidiolic acid
CBD: Cannabidiol
THCA: Delta-9-Tetrahydrocannabinolic acid
THC: Delta-9-Tetrahydrocannabinol
