# Supplementary material for: High-THC Cannabis sativa in a New York greenhouse: yield and economic factors
Source: J Cannabis Res. 2026 May 6;8:85. doi: 10.1186/s42238-026-00429-5 (PMC13374136; doi:10.1186/s42238-026-00429-5)
Supplement: Supplementary file 1 — Supplementary Material 1. Figure S1. Changes in growth parameters over time for autoflower Cannabis sativa plants, analyzed using linear mixed-effects models with repeated measures: (A) height showed a significant change through time (F = 1075.3; P < 0.0001) and all timepoints differ from each other (P<0.0001) except timepoints 4 and 5, (B) stem diameter significantly changed through time (F = 1081.7; P < 0.0001) and all timepoints differ from each other (P<0.0001) except timepoints 4 and 5, and (C) the number of nodes exhibited significant variation over time (F = 483.58; P < 0.0001), with all timepoints differing from each other with a significance of P<0.0001 except timepoints 3 and 4 with a significance of P<0.03. Figure S2. Temporal changes in growth parameters of photoperiod C. sativa plants, analyzed using linear mixed-effects models with repeated measures: (A) height significantly increased over time (F = 477.36; P < 0.0001) with all timepoints differing from each other (P<0.0001), (B) stem diameter significantly increased over time (F = 170.44; P < 0.0001) with all timepoints differing from each other (P<0.0001), and (C) the number of nodes significantly increased over time (F = 116.88; P < 0.0001) with all timepoin. Lines in R code 2113. Figure S3. Growth and biomass traits of photoperiod C. sativa plants during harvest. The varieties planted two weeks earlier (with 11 additional days in the ground; Age 1) showed statistically significant differences during harvest in (A) height (F=8.951, P<0.001), (B) Stem diameter (F= 11.09, P<0.0001), and (C) Number of Nodes (F=6.883, P< 0.005). Figure S4. Estimated Harvest Index based on the lost mass on water from the Autoflower measurements and on the lost mass from stems and twigs after bucking from the Photoperiod measurements. [file 42238_2026_429_MOESM1_ESM.docx]

**Supplementary Material**


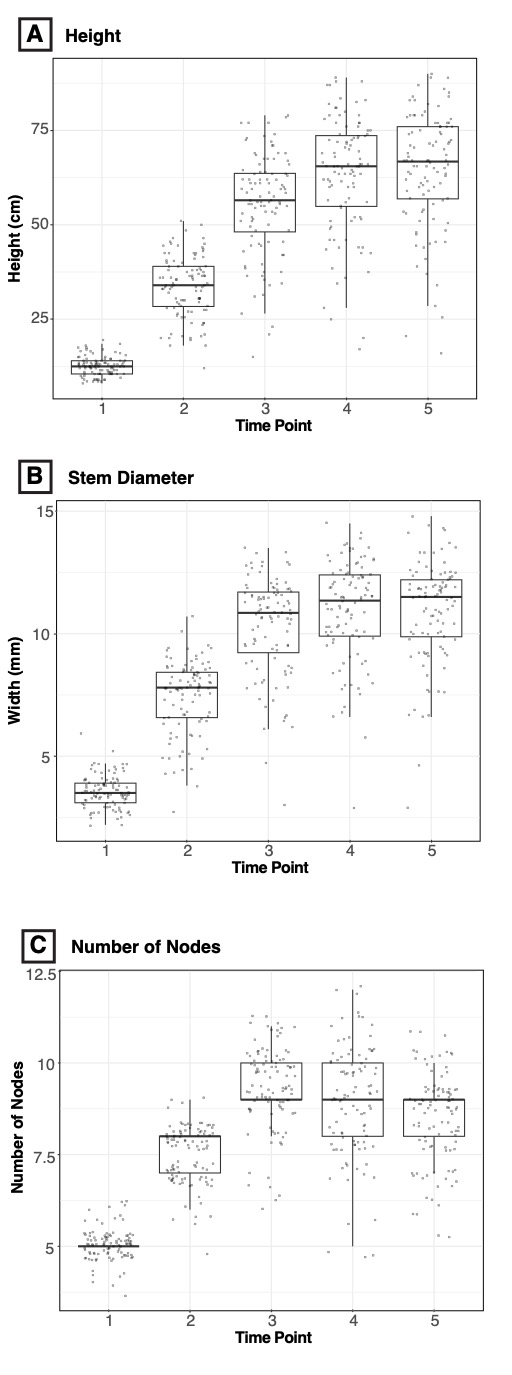


**Figure S1.** Changes in growth parameters over time for autoflower *Cannabis sativa* plants, analyzed using linear mixed-effects models with repeated measures: (A) height showed a significant change through time (F = 1075.3; P < 0.0001) and all timepoints differ from each other (P<0.0001) except timepoints 4 and 5, (B) stem diameter significantly changed through time (F = 1081.7; P < 0.0001) and all timepoints differ from each other (P<0.0001) except timepoints 4 and 5, and (C) the number of nodes exhibited significant variation over time (F = 483.58; P < 0.0001), with all timepoints differing from each other with a significance of P<0.0001 except timepoints 3 and 4 with a significance of P<0.03.


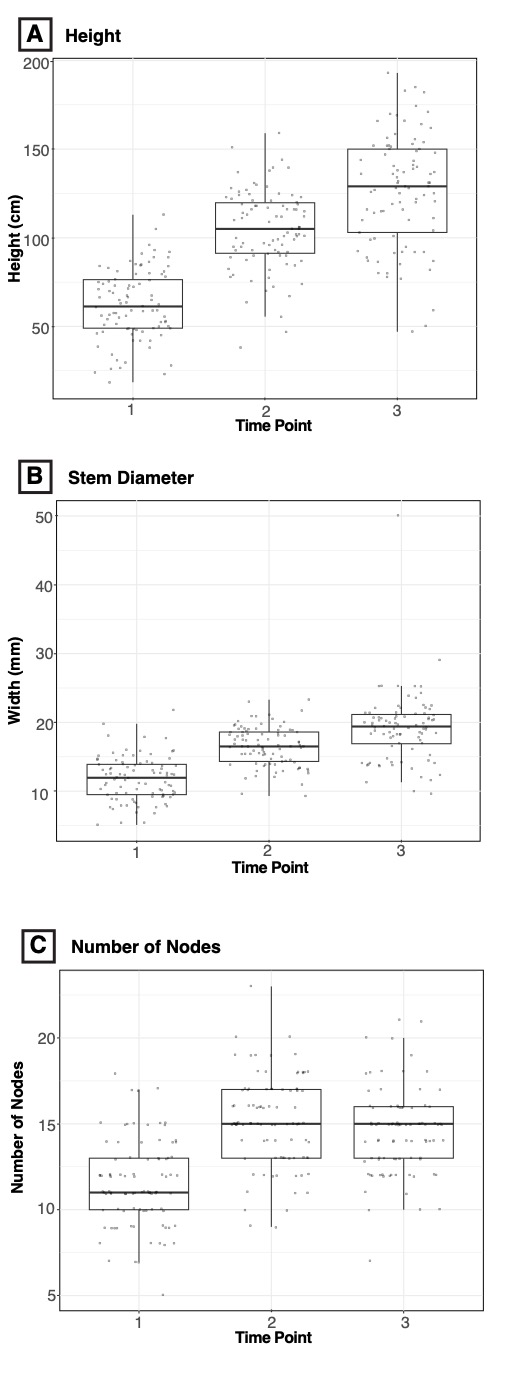


**Figure S2.** Temporal changes in growth parameters of photoperiod *C. sativa* plants, analyzed using linear mixed-effects models with repeated measures: (A) height significantly increased over time (F = 477.36; P < 0.0001) with all timepoints differing from each other (P<0.0001), (B) stem diameter significantly increased over time (F = 170.44; P < 0.0001) with all timepoints differing from each other (P<0.0001), and (C) the number of nodes significantly increased over time (F = 116.88; P < 0.0001) with all timepoin. Lines in R code 2113

**
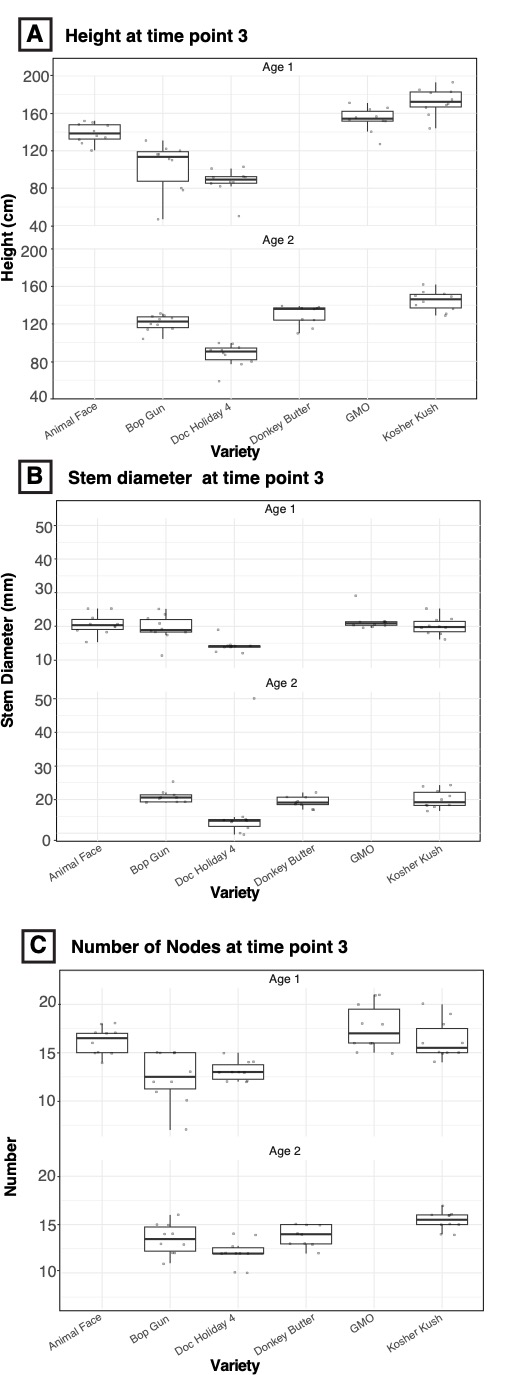
**

**Figure S3.** Growth and biomass traits of photoperiod *C. sativa* plants during harvest. The varieties planted two weeks earlier (with 11 additional days in the ground; Age 1) showed statistically significant differences during harvest in (A) height (F=8.951, P<0.001), (B) Stem diameter (F= 11.09, P<0.0001), and (C) Number of Nodes (F=6.883, P< 0.005).


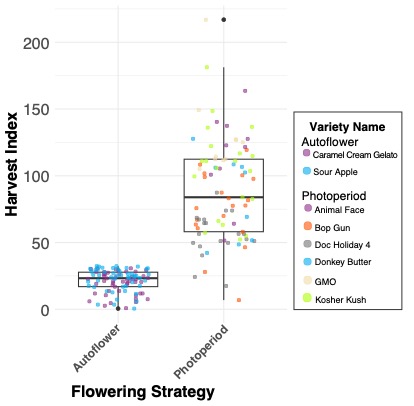


**Figure S4.** Estimated Harvest Index based on the lost mass on water from the Autoflower measurements and on the lost mass from stems and twigs after bucking from the Photoperiod measurements
